# Results of a Stepped-Wedge Cluster-Randomized Implementation Science Trial to Improve Chlorhexidine Gluconate Bathing Compliance

**DOI:** 10.1017/ash.2021.126

**Published:** 2021-07-29

**Authors:** Staci Reynolds, Edward Keating

## Abstract

**Background:** Daily chlorhexidine gluconate (CHG) bathing in intensive care units (ICUs) is widely supported in the literature to decrease risk of central-line-associated infections (CLABSIs). However, adoption of this practice is inconsistent. The primary objective of this implementation science study was to assess the effect of a bathing intervention on compliance with the AHRQ CHG bathing protocol. Secondary objectives were to examine (1) moderating effects of unit characteristics, (2) the intervention effect on nursing staff’s knowledge and perceptions of CHG bathing, and (3) the intervention effect on CLABSI rates. **Methods:** A stepped-wedge cluster-randomized design was used to implement and evaluate the effectiveness of a CHG bathing intervention. At 2 large hospitals, 14 units were clustered into 4 sequences. Units included 9 adult ICUs, 3 pediatric ICUs, 1 pediatric bone marrow transplant unit, and 1 adult hematology-oncology unit. Sequences were enrolled into the active intervention phase over the course of 4 months. The intervention included 2 evidence-based implementation strategies: (1) educational outreach visits and (2) audit and feedback on CHG bathing compliance. Compliance with the CHG bathing processes and daily CHG bathing documentation were assessed. At 12 months after the last enrolled date, booster sessions were completed, and outcomes were assessed for sustainability. **Results:** In models of CHG bathing process compliance, the implementation strategy was significant (b = 6.97; *P* = .009), indicating that compliance was 6.97% higher after the intervention than before. There was a statistically significant improvement in nursing knowledge of CHG bathing (χ^2^ = 9.32, p = .002) and perception of the priority of CHG bathing (t = 2.56; *P* = .01). Daily CHG bathing documentation compliance and CLABSI rates did not significantly improve immediately following the intervention; however, a clinically significant decrease (27.4%) in CLABSI rate was observed. At 12 months after the intervention, improvements in CHG bathing documentation and process outcomes were sustained. There was no change in bathing process compliance after 12 months (b = −0.19; *P* = .87; intercept=96.96, p < .001), and compliance remained high at 96.96%. There was no change in documentation compliance after 12 months (b=3.89, p=.37, intercept=78.72, p < .001), and compliance remained high at 78.72%. After 12 months, CLABSI rates were statistically significantly reduced (b = −0.16; *P* = .009; intercept =1.97, p < .001). **Conclusions:** Using educational outreach visits and audit-and-feedback strategies can improve the adoption of evidence-based CHG bathing practices. CHG bathing—a simple, nurse-driven practice—can make a significant improvement in patient outcomes.

**Funding**: No

**Disclosures:** None

